# The TaERF3-*TaPROT2* Module Enhances Wheat Cadmium Tolerance

**DOI:** 10.3390/plants15121769

**Published:** 2026-06-08

**Authors:** Hong Zhang, Huanqiang Guo, Juncheng Wang, Xiaole Ma, Lirong Yao, Erjing Si, Baochun Li, Yaxiong Meng, Ke Yang, Xunwu Shang, Huajun Wang

**Affiliations:** 1 State Key Lab of Aridland Crop Science, Gansu Key Lab of Crop Improvement and Germplasm Enhancement, Lanzhou 730070, China; 15193116768@163.com (H.Z.); 13051534201@163.com (H.G.); wangjc@gsau.edu.cn (J.W.); maxl@gsau.edu.cn (X.M.); shangxunwu@163.com (X.S.); 2 Department of Crop Genetics and Breeding, College of Agronomy, Gansu Agricultural University, Lanzhou 730070, China; 3 Department of Botany, College of Life Sciences and Technology, Gansu Agricultural University, Lanzhou 730070, China

**Keywords:** *Triticum aestivum*, Cd tolerance, proline transporter, ERF transcription factor, heavy metal detoxification

## Abstract

Cadmium (Cd) toxicity poses a significant threat to crop production and food safety. Although proline is known to enhance plant tolerance to Cd, the molecular mechanisms regulating Cd detoxification through proline accumulation remain unclear. This study identifies the proline transporter *TaPROT2* as a crucial positive regulator of Cd tolerance in wheat. We demonstrate that overexpression of *TaPROT2* directly promotes proline accumulation in transgenic wheat while simultaneously activating the antioxidant enzyme system, thereby reducing both Cd accumulation and translocation. Using electrophoretic mobility shift assays (EMSA), yeast one-hybrid (Y1H) assays, and luciferase reporter assays, we confirmed that TaERF3 directly binds to the GCC-box element in the *TaPROT2* promoter, thereby activating its transcription. Furthermore, overexpression of *TaERF3* enhances the expression of *TaPROT2*, leading to increased proline accumulation and decreased Cd content. In summary, our study reveals a novel TaERF3-*TaPROT2* module that promotes proline accumulation, reduces Cd accumulation, and enhances Cd tolerance, providing a promising target for breeding low-Cd wheat.

## 1. Introduction

Cadmium (Cd), a highly toxic and bioaccumulative heavy metal, poses a significant threat to the global environment due to its inherent mobility and persistence. Human activities such as electroplating, battery manufacturing, and the application of Cd-contaminated phosphate fertilizers and sewage irrigation in agriculture are the primary sources of Cd pollution [1]. Long-term exposure to even low doses of Cd can lead to severe health consequences, including kidney damage, osteoporosis, various cancers (e.g., lung and prostate cancer), and impaired neurodevelopment, particularly in children [2]. Wheat (*Triticum aestivum* L.), a globally important food crop, serves as a primary pathway for Cd exposure. Given the significant risks that Cd poses to agriculture and food safety, there is an urgent need to implement effective prevention and control strategies that integrate molecular breeding with environmental protection.

Proline, a widely recognized compatible solute in plants, serves not only as a fundamental amino acid for protein synthesis but also plays a central regulatory role in plant responses to abiotic stress. Extensive research has demonstrated that under adverse conditions, proline accumulates significantly in plants, mitigating stress-induced damage through various physiological and biochemical pathways, thereby maintaining cellular homeostasis and normal metabolism [3,4,5]. Furthermore, exogenous proline significantly enhances plant tolerance to heavy metals. For instance, exogenous proline has been shown to confer tolerance to Cd stress in *Nicotiana tabacum* [6] and *Vigna radiata* [7].

Proline transporters (PROTs) are essential carriers that facilitate the transmembrane transport of proline, thereby regulating its dynamic distribution across various plant tissues, cells, and organelles [8]. They serve as a critical link between proline synthesis and functional expression, playing an indispensable role in plant responses to abiotic stress [9]. PROTs belong to the amino acid transporter superfamily and can be phylogenetically and functionally categorized into subfamilies, including the major facilitator superfamily (MFS) and amino acid permeases (AAP) [10]. Members of these families exhibit significant variations in localization, substrate specificity, and stress response patterns [11]. Most PROTs are localized in the plasma membrane and are responsible for the long-distance transport of proline between cells, while certain members are situated on organelle membranes, such as the tonoplast and mitochondrial membrane, where they participate in the compartmentalized storage and metabolic regulation of proline [12]. Substrate specificity analyses indicate that the majority of PROTs can specifically transport proline, with some members also capable of mediating the transport of other compatible solutes, such as γ-aminobutyric acid (GABA), collectively enhancing plant stress resistance [13]. In response to heavy metal stress, PROTs enhance plant tolerance to heavy metals by regulating the intracellular distribution of proline. In rice, both OsPROT1 and OsPROT3 are localized to the plasma membrane and specifically mediate the transport of proline and GABA. These genes are significantly induced in the shoot parts of rice under Cd stress [14]. Furthermore, under aluminum (Al) toxicity stress, the expression of PROT genes in pak choi roots is activated, facilitating proline transport to root tips. This process alleviates Al toxicity-induced inhibition of root growth by chelating Al ions in root tip cells and maintaining plasma membrane integrity [15]. In summary, PROTs play a pivotal regulatory role in plant responses to abiotic stresses, such as drought, salinity, and heavy metals, by modulating the spatiotemporal distribution and compartmentalization of proline. Their functionality relies on synergistic interactions with proline biosynthesis and hormonal signaling pathways. Elucidating the mechanisms and regulatory networks of PROTs, along with exploring their potential applications in enhancing crop stress resistance, can provide novel strategies to tackle agricultural challenges posed by abiotic stresses, holding significant theoretical and practical implications.

In this study, we identified a wheat proline transporter, *TaPROT2*, and investigated its role and molecular mechanisms in response to Cd stress. Through yeast one-hybrid (Y1H) assay, electrophoretic mobility shift assay (EMSA), and dual-luciferase reporter gene experiments, we demonstrated that *TaERF3* enhances proline accumulation by promoting the transcription of *TaPROT2*, thereby improving resistance to Cd stress in wheat. Our study establishes the TaERF3-*TaPROT2* regulatory pathway, revealing a novel molecular mechanism by which ERF transcription factors regulate Cd stress tolerance through the modulation of proline accumulation in wheat.

## 2. Results

### 2.1. Exogenous Proline Enhances Cd Tolerance in Wheat

**Figure 1:**
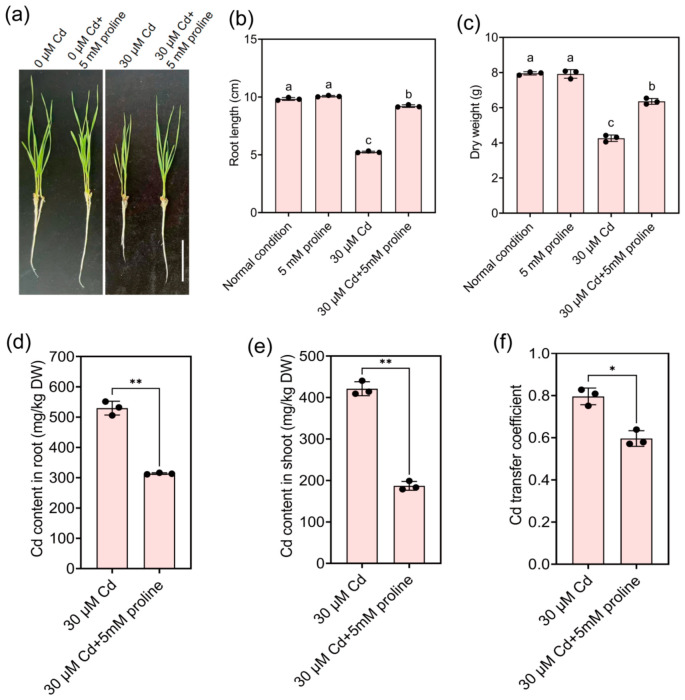
Exogenous proline enhances Cd tolerance in wheat: (**a**) Phenotypes, (**b**) root length, and (**c**) dry weight of wheat seedlings under normal conditions, 5 mM proline, 30 μM Cd, and 5 mM proline + 30 μM Cd. Data are presented as mean ± standard deviation (SD) from three biological replicates (*n* = 3). Statistical significance was determined using a one-way ANOVA followed by Tukey’s HSD post hoc test for multiple comparisons. Different letters above the bars indicate statistically significant differences (*p* < 0.05). Scale bar in (**a**) = 5 cm. (**d**,**e**) Cd contents in root and shoot tissues under 30 μM Cd and 5 mM proline + 30 μM Cd. (**f**) Cd transfer coefficient under 30 μM Cd and 5 mM proline + 30 μM Cd. Data are presented as mean ± standard deviation (SD) from three biological replicates (*n* = 3). Statistical significance was determined using a *t*-test. Asterisks above bars indicate statistically significant differences (* *p* < 0.05; ** *p* < 0.01).

Proline is a crucial metabolite involved in plants’ responses to abiotic stress. Our study demonstrated that the exogenous application of 5 mM proline significantly enhanced the Cd tolerance of wheat (Figure 1a). This enhancement was evidenced by a 49.17% increase in dry weight (Figure 1b), a 75.71% elongation of root length (Figure 1c), a 40.79% reduction in root Cd content (Figure 1d), and a 55.61% decrease in shoot Cd content (Figure 1e). Additionally, the cadmium translocation factor decreased by 25.08% (Figure 1f). These findings indicate that exogenous proline effectively improves Cd tolerance in wheat while simultaneously inhibiting Cd accumulation and translocation within the plants.

### 2.2. Overexpression of TaPROT2 Enhances Cd Tolerance in Wheat

Given that proline can mitigate Cd tolerance in wheat, we analyzed the response of the proline transporter protein to Cd stress and found that *TaPROT2* exhibited a significant response to this stress. After 12 h of Cd treatment, the expression level of *TaPROT2* peaked and subsequently decreased, although it remained elevated. This observation suggests that *TaPROT2* plays a crucial role in the response to Cd stress (Supplementary Figure S1).

**Figure 2:**
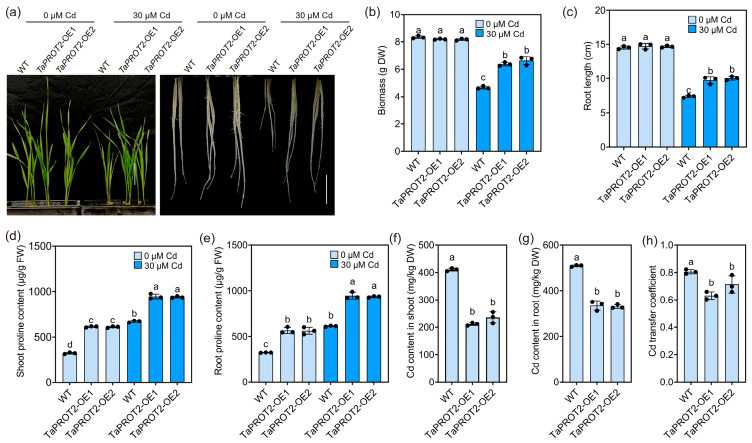
Overexpression of TaPROT2 enhances Cd tolerance in wheat seedlings: (**a**) Phenotypes of WT and TaPROT2-overexpression lines grown for 14 days under normal conditions or exposed to 30 µM Cd. Scale bar = 5 cm. (**b**–**h**) Quantification of dry weight (**b**), root length (**c**), shoot proline content (**d**), root proline content (**e**), shoot Cd content (**f**), root Cd content (**g**), and Cd transfer coefficient (**h**) of WT and TaPROT2-overexpression lines under control and Cd stress conditions. Data are presented as mean ± standard deviation (SD) from three biological replicates (*n* = 3). Statistical significance was determined using a one-way ANOVA followed by Tukey’s HSD post hoc test for multiple comparisons. Different letters above the bars indicate statistically significant differences (*p* < 0.05).

To further investigate the impact of *TaPROT2* on Cd tolerance in plants, we expressed *TaPROT2* in wheat. The CDS of *TaPROT2* was inserted into the pCAMBIA1301 vector under the control of the 35S promoter and subsequently transformed into the wheat cultivar Fielder. We obtained five positive transgenic wheat lines and identified the relative expression levels of *TaPROT2* using qPCR. Ultimately, we selected the two lines with the highest expression levels for subsequent studies (Supplementary Figure S2). Hydroponic analysis revealed that *TaPROT2*-overexpressing seedlings exhibited enhanced tolerance to Cd treatment (Figure 2a), as evidenced by increased seedling dry weight (Figure 2b) and longer primary roots (Figure 2c). These results indicate that *TaPROT2* enhances plant tolerance to Cd. Given that TaPROT2 is involved in proline transport, we measured the proline content in transgenic wheat. The results demonstrated that, under normal growth conditions, the proline content in both the roots and shoots of *TaPROT2*-overexpressing wheat was significantly higher than that in WT plants (Figure 2d,e). Under Cd stress, the proline content in both WT and *TaPROT2*-overexpressing wheat increased significantly; however, the proline content in *TaPROT2*-overexpressing wheat remained significantly higher than that in WT (Figure 2d,e).

### 2.3. Overexpression of TaPROT2 Inhibits Cd Accumulation in Wheat

To clarify the importance of *TaPROT2* in the accumulation of Cd, we subjected both WT and *TaPROT2*-overexpressing wheat plants to 30 µM Cd during the three-leaf stage for a duration of 14 days. Subsequently, we measured the Cd levels in the roots and shoots. The findings indicated that the Cd concentrations in both the roots and shoots of *TaPROT2*-overexpressing wheat were significantly lower than those in the WT (Figure 2f,g). Additionally, the efficiency of translocation from roots to stems was noticeably reduced (Figure 2h). In conclusion, *TaPROT2* enhances Cd tolerance in wheat, likely by facilitating proline accumulation.

### 2.4. Effects of TaPROT2 Overexpression on Antioxidant Enzyme Activities in Seedlings

**Figure 3:**
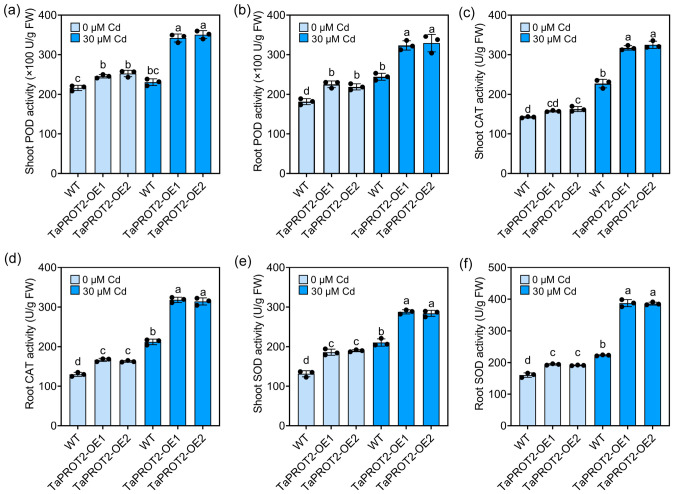
Overexpression of TaPROT2 enhances antioxidant enzyme activities in wheat seedlings: (**a**,**b**) POD, (**c**,**d**) CAT, and (**e**,**f**) SOD activities of WT and TaPROT2-overexpression lines grown for 14 days under normal conditions or exposed to 30 µM Cd. Data are presented as mean ± standard deviation (SD) from three biological replicates (*n* = 3). Statistical significance was determined using a one-way ANOVA followed by Tukey’s HSD post hoc test for multiple comparisons. Different letters above the bars indicate statistically significant differences (*p* < 0.05).

To investigate the regulatory effects of *TaPROT2* gene overexpression on the wheat antioxidant enzyme system and its function in response to Cd stress, we utilized transgenic wheat and WT wheat as experimental materials. After 14 days under normal conditions and Cd stress treatment, we measured the activity changes of POD, CAT, and SOD in root and shoot tissues. The results indicated that in shoot tissues, the activities of POD, CAT, and SOD in transgenic plants were significantly higher than those in WT controls under both normal growth conditions and Cd stress conditions (Figure 3a,c,e). In root tissues, transgenic plants also exhibited significantly enhanced antioxidant enzyme activities, a phenomenon consistently observed under both non-stress and Cd stress conditions (Figure 3b,d,f). The elevated activities of antioxidant enzymes in TaPROT2-overexpressing lines under normal conditions indicate that the accumulation of proline mediated by TaPROT2 primes the antioxidant system, facilitating a more rapid and robust response to subsequent Cd stress. The elevated activities of antioxidant enzymes in *TaPROT2*-overexpressing lines under normal conditions coincide with the increased proline content, reflecting a highly coordinated cellular defense network that may synergistically enhance the plant’s capacity to alleviate subsequent Cd stress.

### 2.5. TaERF3 Promotes TaPROT2 Expression by Directly Binding to Its Promoter

**Figure 4:**
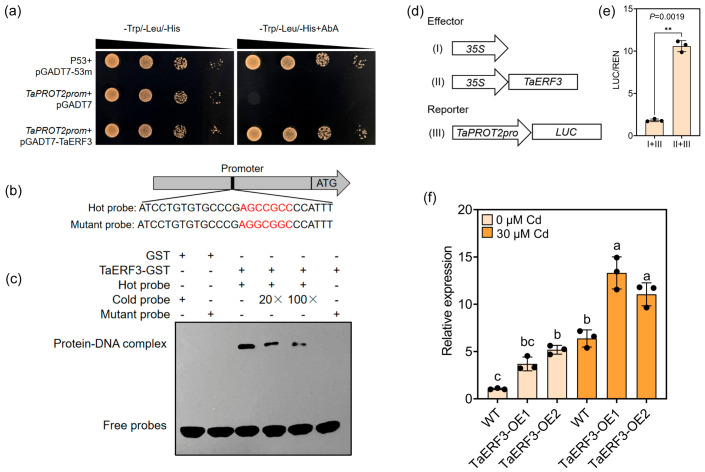
TaERF3 directly binds to the promoter of TaPROT2: (**a**) The yeast one−hybrid (Y1H) assay was carried out to confirm the interaction between TaERF3 and the TaPROT2 promoter. Yeast cells transformed with P53 + pGADT7−53m were used as a positive control. (**b**) Schematic representation of the *TaPROT2* promoter regions. Red color indicates the positions of GCC-box motifs. A core sequence containing AGCCGCC was used as the target probe, with a mutated core sequence containing AGGCGGC as a negative control. (**c**) Electrophoretic mobility shift assay (EMSA) was performed to evaluate the binding of recombinant TaERF3 to biotin-labeled oligonucleotide probes containing the GCC−box motif derived from the TaPROT2 promoter. (**d**) Schematic diagram of the effector and reporter constructs used in dual−luciferase reporter assays. (**e**) Dual−luciferase reporter assays demonstrating the regulatory effect of TaERF3 on the *TaPROT2* promoter in tobacco leaves. Data are presented as mean ± standard deviation (SD) from three biological replicates (*n* = 3). Statistical significance was determined using a *t*-test. Asterisks above bars indicate statistically significant differences (** *p* < 0.01). (**f**) Expression of TaPROT2 in WT and TaERF3-overexpression wheat under normal conditions and Cd stress. Data are presented as mean ± standard deviation (SD) from three biological replicates (*n* = 3). Statistical significance was determined using a one-way ANOVA followed by Tukey’s HSD post hoc test for multiple comparisons. Different letters above the bars indicate statistically significant differences (*p* < 0.05).

Given the critical role of *TaPROT2* in plant responses to Cd stress, our next objective was to identify the upstream transcriptional regulators that directly mediate the transcription of *TaPROT2*. We employed a Y1H library screening to detect transcription factors that interact directly with the *TaPROT2* promoter fragment. Our findings revealed that TaERF3 potentially binds to the *TaPROT2* promoter, and subsequent point-to-point Y1H analysis confirmed the interaction between TaERF3 and the *TaPROT2* promoter (Figure 4a). We identified a GCC-box within the *TaPROT2* promoter that may bind to ERF transcription factors (Figure 4b), after which we synthesized biotin-labeled hot probes. We then conducted competitive EMSA to further investigate the interaction between TaERF3 and the *TaPROT2* promoter. Following incubation of the TaERF3-GST fusion protein with the probe, we observed a significant retardation in the mobility of the probe fragment, while cold competitor probes competed with the hot probe for TaERF3-GST binding activity. Conversely, recombinant TaERF3-GST did not interact with the mutated probe (Figure 4c). To further analyze the transcriptional regulation of *TaPROT2* by TaERF3, we conducted a luciferase reporter assay, which showed that the TaERF3 transcription factor significantly activates the expression of *TaPROT2* (Figure 4d,e). qRT-PCR analysis was conducted to investigate the expression changes of *TaERF3* following Cd treatment over a 72 h time course. The transcription of *TaERF3* was found to be significantly upregulated after Cd application compared to the 0 h control. Its expression steadily increased, reaching a peak at 6 h, and subsequently declined as the treatment duration extended to 72 h. Equivalent expression levels were observed at both 48 and 72 h (Supplementary Figure S3). Furthermore, we generated *TaERF3*-overexpressing wheat lines (Supplementary Figure S4) and analyzed the expression of *TaPROT2* in these lines. The results indicated that overexpression of *TaERF3* significantly increased the expression level of *TaPROT2* (Figure 4f), suggesting that TaERF3 promotes the expression of *TaPROT2*. In summary, TaERF3 directly interacts with the *TaPROT2* promoter and positively regulates *TaPROT2* expression.

### 2.6. Overexpression of TaERF3 Enhances Cd Tolerance in Wheat

**Figure 5:**
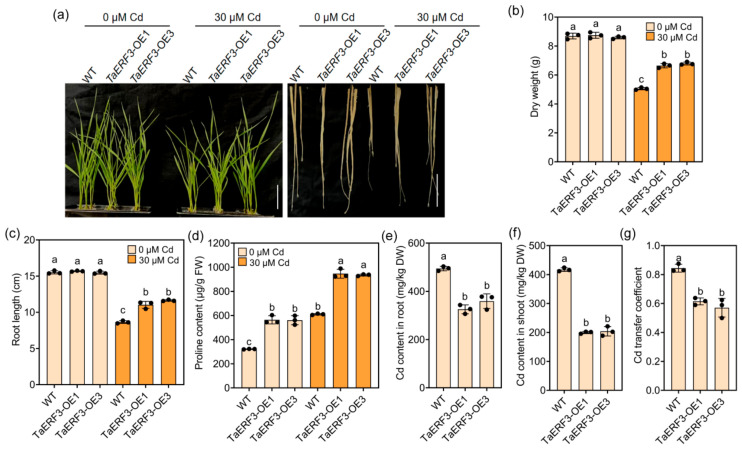
Overexpression of *TaERF3* enhances Cd tolerance in wheat seedlings: (**a**) Phenotypes of WT and *TaERF3*-overexpression lines grown for 14 days under normal conditions or exposed to 30 µM Cd. Scale bar = 5 cm. (**b**–**g**) Quantification of dry weight (**b**), root length (**c**), shoot proline content (**d**), root proline content (**e**), shoot Cd content (**f**), and root Cd content (**g**) of WT and *TaERF3*-overexpression lines under control and Cd stress conditions. Data are presented as mean ± standard deviation (SD) from three biological replicates (*n* = 3). Statistical significance was determined using a one-way ANOVA followed by Tukey’s HSD post hoc test for multiple comparisons. Different letters above the bars indicate statistically significant differences (*p* < 0.05).

To investigate the physiological role of *TaERF3* in response to Cd stress, we constructed *TaERF3*-overexpressing wheat lines in the Fielder background (Supplementary Figure S1). After 14 days of exposure to 30 µM Cd, the *TaERF3* overexpression lines exhibited a pronounced growth-enhanced phenotype compared to WT plants (Figure 5a). To quantify these observations, we measured plant biomass and root length under control and Cd-treated conditions. Under normal conditions, no statistically significant differences were detected between WT and *TaERF3*-overexpressing lines for these parameters (Figure 5b,c). In contrast, under Cd stress, *TaERF3*-overexpressing plants exhibited significantly increased plant biomass and root length compared to WT plants (Figure 5b,c). Furthermore, proline content was significantly higher in *TaERF3*-overexpressing plants under normal conditions and Cd stress compared to WT (Figure 5d). These results demonstrate that *TaERF3* overexpression significantly enhances endogenous proline levels in wheat, thereby improving its tolerance to Cd stress.

### 2.7. Overexpression of TaERF3 Inhibits Cd Accumulation in Wheat

To elucidate the role of *TaERF3* in Cd accumulation, we treated both WT and *TaERF3*-overexpressing wheat plants at the three-leaf stage with 30 µM Cd for a duration of 14 days. Compared to the WT plants, the Cd concentration in the roots decreased by 34.2% (Figure 5e), whereas the concentration in the shoots decreased by 52.1% (Figure 5f). Furthermore, an analysis of Cd translocation efficiency from roots to shoots indicated that the overexpression of *TaERF3* significantly inhibited the Cd transfer coefficient in wheat (Figure 5g). Thus, *TaERF3* overexpression not only suppressed Cd accumulation in wheat but also inhibited Cd translocation.

### 2.8. Effects of TaERF3 Overexpression on Antioxidant Enzyme Activities in Seedlings

**Figure 6:**
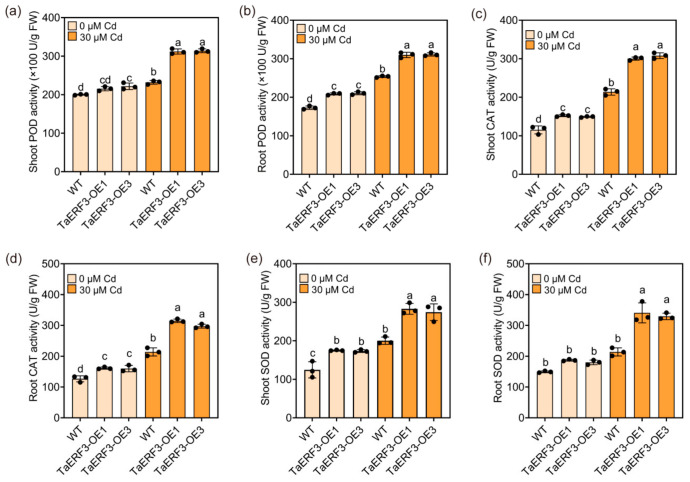
Overexpression of TaERF3 enhances antioxidant enzyme activities in wheat seedlings: (**a**,**b**) POD, (**c**,**d**) CAT, and (**e**,**f**) SOD activities of WT and TaERF3-overexpression lines grown for 14 days under normal conditions or exposed to 30 µM Cd. Data are presented as mean ± standard deviation (SD) from three biological replicates (*n* = 3). Statistical significance was determined using a one-way ANOVA followed by Tukey’s HSD post hoc test for multiple comparisons. Different letters above the bars indicate statistically significant differences (*p* < 0.05).

As shown in Figure 6, the overexpression of *TaERF3* significantly altered the activities of POD, CAT, and SOD in both roots and shoots compared to the WT group. After 14 days of Cd treatment, the activities of POD, CAT, and SOD in the shoots exhibited significant alterations. Under normal conditions, the activities of these enzymes in the shoots of *TaERF3*-overexpressing wheat were markedly elevated. Similarly, under Cd stress, the activities of these enzymes were also significantly higher than those observed in the WT group (Figure 6a,c,e). At the root level, under normal conditions, the overexpression of *TaERF3* significantly increased the activities of POD and CAT in wheat, while no significant change was noted in SOD activity, despite the average value being higher than that of the WT. However, under Cd stress, the activities of POD, CAT, and SOD in *TaERF3*-overexpressing wheat were all significantly elevated compared to WT (Figure 6b,d,f). This finding indicates that the overexpression of *TaERF3* can effectively enhance the enzyme-mediated ROS-scavenging capacity under Cd stress.

## 3. Discussion

Plants have evolved highly sophisticated and interconnected physiological and molecular strategies to limit Cd uptake and enhance tolerance under toxicity stress. To limit Cd entry, the root cell wall first immobilizes Cd ions via adsorption, while root exudates, such as organic acids, chelate extracellular Cd, thereby reducing its bioavailability. Competitive inhibition by mineral nutrients further prevents Cd from entering root cells through shared transporters. Once Cd penetrates the cells, plants activate detoxification pathways. Phytochelatins and metallothioneins bind to Cd in the cytoplasm, forming stable complexes that are subsequently sequestered into the vacuole to avert damage to organelles. Concurrently, the antioxidant system, comprising SOD, CAT, and glutathione, effectively scavenges the excessive reactive oxygen species (ROS) induced by Cd toxicity, thus maintaining cellular redox homeostasis. These avoidance and detoxification mechanisms operate synergistically to mitigate Cd toxicity [3,4,6].

This study demonstrates that Cd significantly inhibits wheat growth. However, the addition of 5 mM exogenous proline enhances antioxidant enzyme activity, thereby improving the tolerance of wheat to Cd (Figure 1a–c). Previous research has established that exogenous proline can protect photosynthetic pigments, maintain photosynthetic efficiency, and preserve cell viability, collectively alleviating Cd toxicity in plants [16,17]. Cd stress typically results in the excessive accumulation of ROS, which disrupts cell elongation and division, ultimately inhibiting plant growth and development [18,19]. The mechanism through which exogenous proline mitigates Cd stress may involve its capacity to scavenge free radicals and maintain cellular redox homeostasis, thereby safeguarding the integrity of subcellular structures [20]. Consequently, the extent of growth inhibition is often utilized as a key indicator for assessing Cd toxicity [21,22]. This study further confirms that exogenous proline significantly promotes root elongation and increases the dry weight of wheat seedlings under Cd stress (Figure 1b,c), thereby enhancing the plant’s tolerance to Cd.

Previous studies have demonstrated that exogenous proline treatment can reduce Cd content in both shoot and root tissues of various plants, including *Brassica juncea*, tobacco, and olive [6,17,20]. Notably, exogenous proline significantly decreased the Cd translocation factor [17], indicating an inhibition of Cd translocation to the shoot. Proline can form complexes with Cd, and exogenous proline enhances the levels of glutathione and phytochelatins, both of which serve as important Cd chelators. Upon binding with Cd in the cytoplasm, phytochelatins (PCs) are typically transported to vacuoles for storage, thereby reducing Cd translocation to the shoots [16]. This study also found that exogenous proline treatment significantly decreased Cd accumulation in wheat roots and shoots, as well as the translocation factor, further illustrating its inhibitory effect on Cd migration within the plant (Figure 1d,e). Notably, the concentration of exogenous proline utilized in this study (5 mM) functions primarily as a physiological signal and cell protectant rather than a bulk osmolyte, which typically requires much higher concentrations. Therefore, the observed mitigation of Cd toxicity at this physiological dosage is highly specific to proline-mediated responses rather than non-specific osmotic or nutritional effects. In summary, exogenous proline effectively mitigates Cd toxicity in wheat and plays a crucial role in limiting Cd accumulation and translocation.

The PROT protein is a crucial molecule in regulating the spatial distribution of proline, which directly influences the efficiency of plant stress responses by mediating proline homeostasis across cells and subcellular compartments [23]. Studies have identified four genes (*SlPROT1*–*SlPROT4*) within the PROT family in tomato; their promoter regions are enriched with binding sites for stress-responsive transcription factors, including MYB, bZIP, and WRKY. This indicates that their expression may be finely regulated by various abiotic stress signaling pathways [24]. Notably, *SlPROT1* and *SlPROT2* exhibited significant upregulation under drought conditions, while *SlPROT3* and *SlPROT4* displayed tissue-specific expression patterns [24]. In cabbage, six *PROT* genes were identified, and under heat stress, *BchPROT1* and *BchPROT4* were upregulated, whereas *BchPROT3* and *BchPROT6* were downregulated [23]. This suggests that different *PROT* members may respond coordinately to environmental stresses through functional differentiation.

Proline, a small molecule metabolite with osmoregulatory and ROS scavenging functions, plays a crucial role in plant responses to drought, salinity, low temperature, and heavy metal stress [25]. Its synergistic effects with the antioxidant enzyme system have emerged as a significant research direction in understanding plant stress tolerance mechanisms [26]. Studies indicate that proline accumulation not only helps maintain cellular osmotic balance but also alleviates oxidative damage by scavenging ROS, a process closely linked to the regulatory activity of antioxidant enzymes such as SOD, CAT, and POD [27]. For instance, under drought stress, plants enhance proline synthesis to stabilize cell membrane structures while simultaneously upregulating the activity of enzymes like SOD and CAT to convert superoxide anions (O_2_^•−^) and hydrogen peroxide (H_2_O_2_) into non-toxic or less toxic substances, thereby forming a dual defense system [28]. In transgenic tobacco, heterologously accumulated proline not only alleviates arsenic-induced photosynthetic inhibition and oxidative damage but also significantly reduces malondialdehyde (MDA) content by activating antioxidant enzyme systems such as SOD and APX [29]. This indicates that proline redistribution mediated by PROT may enhance plant tolerance to abiotic stress through dual pathways of osmotic protection and redox homeostasis. In this study, the overexpression of *TaPROT2* significantly improved Cd tolerance and reduced Cd accumulation in wheat, accompanied by an increase in proline content within the plants (Figure 2). Further enzyme activity assays revealed that the rise in proline levels coincided with markedly elevated activities of POD, CAT, and SOD under Cd stress. While these data establish a strong statistical and physiological association rather than a direct linear causation, they indicate that the TaERF3-*TaPROT2* module contributes to Cd tolerance through the intimate coordination of proline homeostasis and cellular redox maintenance.

Transcriptional regulation is a critical mechanism underlying plant responses to abiotic stress. The ERF family, a prominent member of the AP2/ERF superfamily, plays a vital role in enhancing plant stress tolerance by regulating the expression of downstream target genes [30]. Research has shown that ERF transcription factors recognize and bind to specific GCC-box sequences through their conserved AP2/ERF domain. This interaction activates or suppresses the expression of a range of stress-responsive genes, thereby improving plant tolerance to abiotic stress [31]. For example, the wild soybean-derived *GsERF1* significantly upregulates ethylene biosynthesis genes, including ACS4/5/6, via the ethylene signaling pathway under aluminum stress, while also modulating the expression of ABA signaling marker genes *ABI1*/*2*/*4*/*5* [32]. Furthermore, the pepper gene *CaERF14* markedly enhances salt and drought stress resistance in transgenic tobacco by activating the antioxidant defense system and promoting the expression of ROS-scavenging-related genes [33]. Our findings indicate that the transcription factor TaERF3 binds to the GCC-box within the promoter of *TaPROT2*, thereby positively regulating the expression of *TaPROT2* (Figure 4). Furthermore, the overexpression of *TaERF3* enhances proline content and antioxidant enzyme activity in transgenic wheat, which collectively improve Cd tolerance in wheat (Figure 5 and Figure 6).

In summary, we have identified a novel regulatory pathway for Cd tolerance in wheat. The transcription factor TaERF3 modulates the expression of *TaPROT2*, which increases proline content, enhances antioxidant enzyme activity, reduces Cd accumulation, and ultimately improves the Cd tolerance of wheat. It is essential to recognize that heavy metal detoxification in plants constitutes a multifaceted network. While our current study highlights the importance of the proline pathway mediated by the TaERF3-TaPROT2 module, we cannot entirely dismiss the potential involvement of alternative mechanisms. As an ERF transcription factor, TaERF3 may broadly regulate downstream targets associated with direct metal transport systems, sequestration pathways, or vacuolar compartmentalization, independent of proline accumulation. Likewise, *TaPROT2* may exhibit unrecognized substrate affinities or indirectly influence cellular metal distribution. These alternative or parallel detoxification pathways represent a promising area of research and will be a primary focus of our future investigations. However, this study was conducted using hydroponic seedlings. Further field trials are necessary to evaluate the agronomic performance and grain Cd accumulation of TaPROT2 or TaERF3 overexpression lines under natural conditions.

## 4. Conclusions

This study reveals a critical regulatory module involving TaERF3 and TaPROT2 that positively modulates Cd tolerance in wheat. Exogenous proline effectively mitigates Cd-induced growth inhibition, reduces Cd accumulation, and enhances antioxidant enzyme activities. Functional analyses demonstrate that the proline transporter *TaPROT2* functions as a key positive regulator, with its overexpression significantly increasing endogenous proline levels, strengthening antioxidant defenses, and restricting Cd uptake and translocation. Mechanistically, the transcription factor TaERF3 directly binds to the GCC-box motif in the *TaPROT2* promoter, thereby activating its transcription. Consistently, *TaERF3* overexpression promotes *TaPROT2* expression, elevates proline accumulation, and confers enhanced Cd tolerance. Collectively, these findings define a novel regulatory pathway for Cd detoxification and provide promising genetic resources for the molecular breeding of low-Cd wheat.

## 5. Materials and Methods

### 5.1. Generation of Wheat Genetic Materials and Plant Growth Conditions

To generate wheat overexpression lines of *TaPROT2* and *TaERF3*, we cloned the full-length coding sequences (CDS) of these genes and inserted them into the pCAMBIA1301 vector, resulting in the construction of recombinant plasmids 35S:*TaPROT2* and 35S:*TaERF3*. Following the method previously described [34], these recombinant plasmids were transformed into the *Agrobacterium tumefaciens* strain EHA105. Subsequently, overexpression lines were obtained through *Agrobacterium*-mediated infiltration of the shoot apex in wheat. Briefly, clean shoot apices from 2-day-old seedlings were inoculated with *Agrobacterium* (OD = 0.5). After co-cultivation at 4 °C in the dark for 14 days, the resulting plants were moved to soil, and positive transformants were validated by PCR. To assess the impact of Cd stress on plant phenotypes, seeds of both wild-type (WT) and transgenic wheat were surface-sterilized and subsequently germinated in a controlled growth chamber. All seedlings were cultivated under a 16 h light/8 h dark photoperiod at a constant temperature of 25 °C during the light period and 22 °C during the dark period, with relative humidity maintained at 60–70%. Seedlings at the three-leaf stage were divided into two groups and exposed to treatments with 0 µM (control) and 30 µM CdCl_2_, respectively. The Cd-containing nutrient solution was also refreshed every day, and all treatments lasted for 14 days.

### 5.2. Gene Expression Analysis

Wheat samples that had been processed were quickly frozen using liquid nitrogen and stored at −80 °C until RNA extraction was performed. Total RNA was extracted from the frozen tissue samples using the RNeasy Plant Mini Kit (Qiagen, Hilden, Germany). First-strand cDNA was generated from 1 μg of the isolated total RNA with the HiScript 1st Strand cDNA Synthesis Kit (Vazyme, Nanjing, China). Real-time quantitative PCR (RT-qPCR) was conducted on the Bio-Rad CFX96™ using ChamQ SYBR Color qPCR Master Mix (Vazyme, Nanjing, China). The *Taactin* gene served as the internal reference gene for normalizing expression levels, and relative expression was determined by the 2^−ΔΔCt^ method.

### 5.3. Analysis of Phenotypic, Biochemical, and Physiological Indicators

Plant phenotypes were recorded through photographs, and plant height and root length were measured. Biomass was determined by drying the samples at 80 °C until a constant weight was achieved. The Cd content was quantified using inductively coupled plasma mass spectrometry (ICP-MS, Thermo Fisher Scientific iCAP-Q, Thermo Fisher Scientific, Bremen, Germany), as described previously [35]. The activities of superoxide dismutase (SOD), peroxidase (POD), and catalase (CAT) were measured using the SOD assay kit (BC0170, Solarbio, Beijing, China), the POD assay kit (BC0090, Solarbio, Beijing, China), and the CAT assay kit (BC0200, Solarbio, Beijing, China), respectively.

### 5.4. Yeast One-Hybrid (Y1H) Assay

A cDNA library derived from wheat roots subjected to Cd stress was utilized for Y1H screening. To create the bait vector for the Y1H assay, the promoter region of *TaPROT2* was synthesized (Sangon Biotech, Shanghai, China) and subsequently inserted into the pAbAi vector (Clontech, Mountain View, CA, USA) using standard molecular cloning techniques. Following this, the full-length coding sequence of *TaERF3* was ligated into the prey vector pGADT7 (Clontech, Mountain View, CA, USA). The resulting recombinant plasmids were then introduced into the Y1H Gold strain. The evaluation of DNA-protein interactions was performed by monitoring the growth of co-transformants on synthetic dextrose plates that lacked leucine but contained aureobasidin A (AbA).

### 5.5. Electrophoretic Mobility Shift Assay (EMSA)

The open reading frame (ORF) of *TaERF3* was cloned into the pGEX-4T-1 vector. The expression and purification of the recombinant GST-TaERF3 fusion protein were carried out as previously described [36]. For the DNA transcription factor binding assay, a 27 bp sequence containing the GCC box from the *TaPROT2* promoter was synthesized and biotin-labeled at the 5′ end (Sangon Biotech, Shanghai, China). Additionally, a GCC box mutant sequence was synthesized to serve as a cold probe. EMSA was conducted using the Chemiluminescent EMSA Kit (Thermo Fisher Scientific, Waltham, MA, USA) in accordance with the manufacturer’s instructions.

### 5.6. Luciferase Reporter Gene Assay

The promoter region of *TaPROT2* was cloned into the pGreenII0800-LUC vector, resulting in the creation of the *TaPROT2pro*::LUC construct. Additionally, the ORF of *TaERF3* was cloned into the pGreenII 62-SK vector to produce the 35S::*TaERF3* construct. These constructs were subsequently introduced into the *Agrobacterium tumefaciens* strain EHA105, which was utilized to infiltrate tobacco leaves. A luciferase reporter gene assay was conducted using the Luciferase Reporter Gene Assay Kit (Promega, Madison, WI, USA). Firefly luciferase activity was normalized to Renilla luciferase activity as an internal control.

### 5.7. Statistical Analysis

Each experiment was conducted independently three times, with each sample also being tested independently three times. The data were analyzed using one-way analysis of variance (ANOVA), followed by the post hoc Tukey honestly significant difference (HSD) test. Paired *t*-tests were used for matched samples and unpaired *t*-tests for independent samples.

## Data Availability

The data presented in this study are available upon request from the corresponding author.

## Supplementary Materials

The following supporting information can be downloaded at: https://www.mdpi.com/article/10.3390/plants15121769/s1, Figure S1: Relative expression levels of *TaPROT2* in wheat after Cd stress treatment at different time points (0, 1, 3, 6, 12, 24, 48 and 72 h); Figure S2: Analysis of the expression of *TaPROT2* in WT and *TaPROT2*-overexpression wheat; Figure S3: Relative expression levels of *TaERF3* in wheat after Cd stress treatment at different time points (0, 1, 3, 6, 12, 24, 48 and 72 h); Figure S4: Analysis of the Expression of *TaERF3* in WT and *TaERF3*-overexpression wheat; Table S1: Primes and probes used in this study.

## References

[1] Jin L., Li C., Addou A.M., Zhang S., Li H. (2026). Global heavy metal-antibiotic co-pollution: Distribution, ARG co-selection, toxic synergism, and AOPs-mediated remediation with focus on non-radical pathways. J. Hazard. Mater..

[2] Rasin P., Ashwathi A.V., Basheer S.M., Haribabu J., Santibanez J.F., Garrote C.A., Arulraj A., Mangalaraja R.V. (2025). Mitigating Cadmium Accumulation in Plants: Mechanisms and Regulation of Tolerance. J. Hazard. Mater. Adv..

[3] Limami A.M., Glévarec G., Ricoult C., Cliquet J.B., Planchet E. (2008). Concerted modulation of alanine and glutamate metabolism in young Medicago truncatula seedlings under hypoxic stress. J. Exp. Bot..

[4] Liu Y., Shi Q., Cao H., Ma Q., Nian H., Zhang X. (2020). Heterologous expression of a *Glycine soja* C2H2 zinc finger gene improves aluminum tolerance in *Arabidopsis*. Int. J. Mol. Sci..

[5] Lourkisti R., Froelicher Y., Herbette S., Morillon R., Tomi F., Gibernau M., Giannettini J., Berti L., Santini J. (2020). Triploid citrus genotypes have a better tolerance to natural chilling conditions of photosynthetic capacities and specific leaf volatile organic compounds. Front. Plant Sci..

[6] Islam M.M., Hoque M.A., Okuma E., Banu M.N., Shimoishi Y., Nakamura Y., Murata Y. (2009). Exogenous proline and glycinebetaine increase antioxidant enzyme activities and confer tolerance to cadmium stress in cultured tobacco cells. J. Plant Physiol..

[7] Hossain M.A., Hasanuzzaman M., Fujita M. (2010). Up-regulation of antioxidant and glyoxalase systems by exogenous glycinebetaine and proline in mung bean confer tolerance to cadmium stress. Physiol. Mol. Biol. Plants.

[8] Lehmann S., Gumy C., Blatter E. (2011). In planta function of compatible solute transporters of the AtProT family. J. Exp. Bot..

[9] Wang H., Tang X., Wang H., Shao H.B. (2015). Proline accumulation and metabolism-related genes expression profiles in Kosteletzkya virginica seedlings under salt stress. Front. Plant Sci..

[10] Li F., Dong C., Yang T., Bao S., Fang W., Lucas W.J., Zhang Z. (2021). The tea plant CsLHT1 and CsLHT6 transporters take up amino acids, as a nitrogen source, from the soil of organic tea plantations. Hortic. Res..

[11] Li F., Dong C., Yang T., Ma J., Zhang S., Wei C., Wan X., Zhang Z. (2019). Seasonal theanine accumulation and related gene expression in the roots and leaf buds of tea plants (*Camellia sinensis* L.*)*. Front. Plant Sci..

[12] Zdunek-Zastocka E., Grabowska A., Michniewska B., Orzechowski S. (2021). Proline concentration and its metabolism are regulated in a leaf age dependent manner but not by abscisic acid in pea plants exposed to cadmium stress. Cells.

[13] Wipf D., Loqué D., Lalonde S., Frommer W.B. (2012). Amino acid transporter inventory of the Selaginella genome. Front. Plant Sci..

[14] Lin T., Sun L., Gong H., Wang Y., Liu L., Zhao Z., Jiang L., Wan J. (2019). *OsProT1* and *OsProT3* function to mediate proline- and γ-aminobutyric acid-specific transport in yeast and are differentially expressed in rice (*Oryza sativa* L.). Rice Sci..

[15] Chauhan D.K., Yadav V., Vaculík M., Gassmann W., Pike S., Arif N., Singh V.P., Deshmukh R., Sahi S., Tripathi D.K. (2021). Aluminum toxicity and aluminum stress-induced physiological tolerance responses in higher plants. Crit. Rev. Biotechnol..

[16] Khalid M., Rehman H.M., Ahmed N., Nawaz S., Saleem F., Ahmad S., Uzair M., Rana I.A., Atif R.M., Zaman Q.U. (2022). Using exogenous melatonin, glutathione, proline, and glycine betaine treatments to combat abiotic stresses in crops. Int. J. Mol. Sci..

[17] Wang Y., Tan P., Chang L., Yue Z., Zeng C., Li M., Liu Z., Dong X., Yan M. (2022). Exogenous proline mitigates toxic effects of cadmium via the decrease of cadmium accumulation and reestablishment of redox homeostasis in Brassica juncea. BMC Plant Biol..

[18] Cuypers A., Vanbuel I., Iven V., Kunnen K., Vandionant S., Huybrechts M., Hendrix S. (2023). Cadmium-induced oxidative stress responses and acclimation in plants require fine-tuning of redox biology at subcellular level. Free Radic. Biol. Med..

[19] Zhang G., Yang J., Zhang M., Li Q., Wu Y., Zhao X., Zhang H., Wang Y., Wu J., Wang W. (2021). Wheat TaPUB1 regulates Cd uptake and tolerance by promoting the degradation of TaIRT1 and TaIAA17. J. Agric. Food Chem..

[20] Zouari M., Ben Ahmed C., Zorrig W., Elloumi N., Rabhi M., Delmail D., Ben Rouina B., Labrousse P., Ben Abdallah F. (2016). Exogenous proline mediates alleviation of cadmium stress by promoting photosynthetic activity, water status and antioxidative enzymes activities of young date palm (*Phoenix dactylifera* L.). Ecotoxicol. Environ. Saf..

[21] Hu S., Chen J., Wang H., Ji E., Su X., Zhu M., Xiang X., Gong L., Zhou Q., Xiao X. (2024). The transcription factor OsNAC5 regulates cadmium accumulation in rice. Ecotoxicol. Environ. Saf..

[22] Li L., Mao D., Sun L., Wang R., Tan L., Zhu Y., Huang H., Peng C., Zhao Y., Wang J. (2022). CF1 reduces grain-cadmium levels in rice (*Oryza sativa*). Plant J..

[23] Tian J., Chang K., Lei Y., Li S., Wang J., Huang C., Zhong F. (2023). Genome-wide identification of proline transporter gene family in non-heading Chinese cabbage and functional analysis of BchProT1 under heat stress. Int. J. Mol. Sci..

[24] Akbudak M.A., Filiz E. (2020). Genome-wide investigation of proline transporter (ProT) gene family in tomato: Bioinformatics and expression analyses in response to drought stress. Plant Physiol. Biochem..

[25] Hu T., Wang Y., Wang Q., Dang N., Wang L., Liu C., Zhu J., Zhan X. (2019). The tomato 2-oxoglutarate-dependent dioxygenase gene SlF3HL is critical for chilling stress tolerance. Hortic. Res..

[26] Zulfiqar F., Ashraf M. (2022). Proline alleviates abiotic stress induced oxidative stress in plants. J. Plant Growth Regul..

[27] Guo S., Ma X., Cai W., Wang Y., Gao X., Fu B., Li S. (2022). Exogenous proline improves salt tolerance of alfalfa through modulation of antioxidant capacity, ion homeostasis, and proline metabolism. Plants.

[28] Ghaffari H., Tadayon M.R., Nadeem M., Cheema M., Razmjoo J. (2019). Proline-mediated changes in antioxidant enzymatic activities and the physiology of sugar beet under drought stress. Acta Physiol. Plant..

[29] Adamipour N., Nazari F., Nalousi A.M., Teixeira da Silva J.A. (2025). Evaluation of the molecular mechanism underlying proline metabolic and catabolic pathways and some morpho-physiological traits of tobacco (*Nicotiana tabacum* L.) plants under arsenic stress. BMC Plant Biol..

[30] Zhang J., Liao J., Ling Q., Xi Y., Qian Y. (2022). Genome-wide identification and expression profiling analysis of maize AP2/ERF superfamily genes reveal essential roles in abiotic stress tolerance. BMC Genom..

[31] Chen Q., Li N., Cui X., Ge F. (2025). AP2/ERF transcription factors regulate the biosynthesis of terpenoids, phenolics, and alkaloids in plants. Hortic. Res..

[32] Li L., Li X., Yang C., Cheng Y., Cai Z., Nian H., Ma Q. (2022). GsERF1 enhances Arabidopsis thaliana aluminum tolerance through an ethylene-mediated pathway. BMC Plant Biol..

[33] Feng D., He S., Chung J.P. (2025). Isolation of the AP2/ERF transcription factor *CaERF14* in pepper and functional characterization under salinity and dehydration stress. Sci. Rep..

[34] Tian H., Mu Y., Yang S., Zhang J., Yang X., Zhang Q., Geng G., Zhang S. (2024). ATAC sequencing and transcriptomics reveal the impact of chromatin accessibility on gene expression in Tritipyrum under salt-stress conditions. Environ. Exp. Bot..

[35] Chen K., Mou P., Zhu A., Chen P., Chen J., Gao G., Wang X., Feng X., Yu C. (2023). A comparative study of different methods for the determination of cadmium in various tissues of ramie (*Boehmeria nivea* L.). Environ. Monit. Assess..

[36] Tougane K., Komatsu K., Bhyan S.B., Sakata Y., Ishizaki K., Yamato K.T., Kohchi T., Takezawa D. (2010). Evolutionarily conserved regulatory mechanisms of abscisic acid signaling in land plants: Characterization of ABSCISIC ACID INSENSITIVE1-like type 2C protein phosphatase in the liverwort Marchantia polymorpha. Plant Physiol..

