# Supplementary material for: The TaERF3-TaPROT2 Module Enhances Wheat Cadmium Tolerance
Source: Plants (Basel). 2026 Jun 8;15(12):1769. doi: 10.3390/plants15121769 (PMC13306912; doi:10.3390/plants15121769)
Supplement: Supplementary file 1 [file plants-15-01769-s001.zip › Supplementary Figures.pdf]

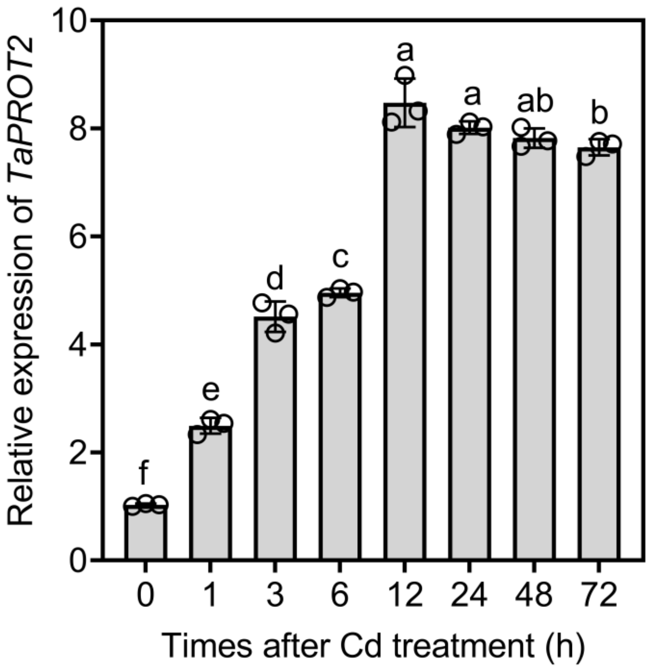

Supplementary Figure S1 Relative expression levels of *TaPROT2* in wheat after Cd stress treatment at different time points (0, 1, 3, 6, 12, 24, 48 and 72 h). Data are presented as mean ± standard deviation (SD) from three biological replicates (n = 3). Statistical significance was determined using a one-way ANOVA followed by Tukey's HSD post-hoc test for multiple comparisons. Different letters above the bars indicate statistically significant differences ($p < 0.05$).

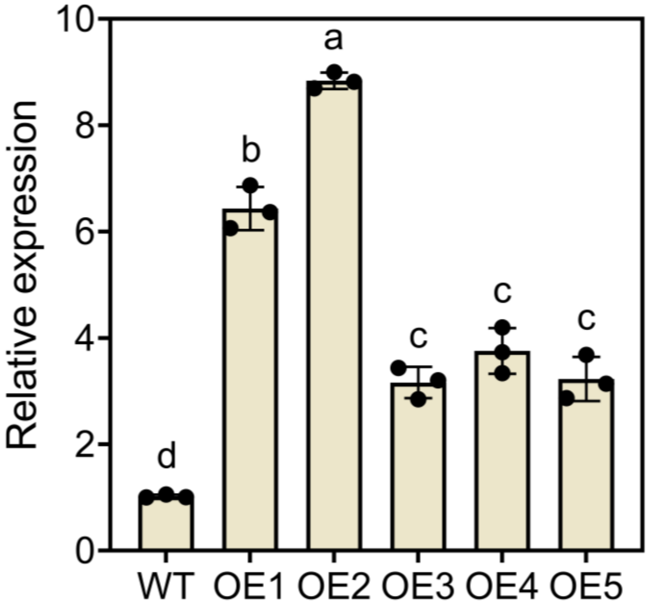

Supplementary Figure S2 Analysis of the Expression of *TaPROT2* in WT and *TaPROT2*-overexpression wheat. Data are presented as mean ± standard deviation (SD) from three biological replicates (n = 3). Statistical significance was determined using a one-way ANOVA followed by Tukey's HSD post-hoc test for multiple comparisons. Different letters above the bars indicate statistically significant differences (p < 0.05).

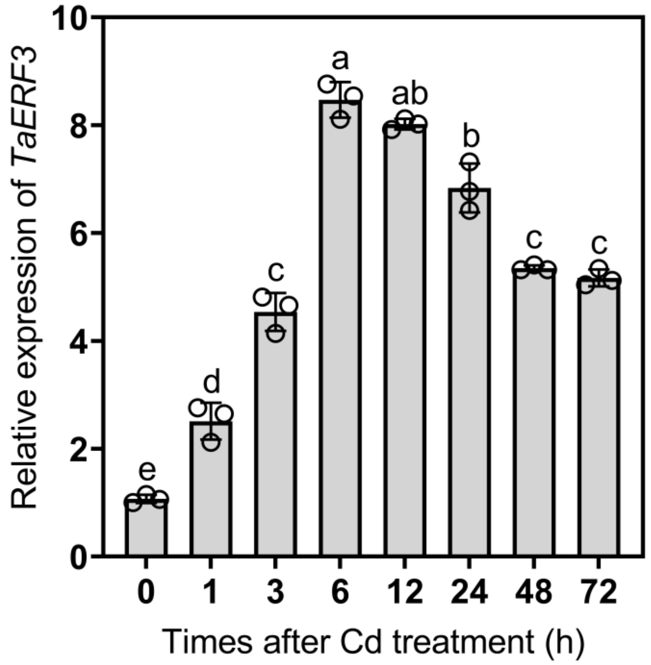

Supplementary Figure S3 Relative expression levels of *TaERF3* in wheat after Cd stress treatment at different time points (0, 1, 3, 6, 12, 24, 48 and 72 h). Data are presented as mean ± standard deviation (SD) from three biological replicates (n = 3). Statistical significance was determined using a one-way ANOVA followed by Tukey's HSD post-hoc test for multiple comparisons. Different letters above the bars indicate statistically significant differences ($p < 0.05$).

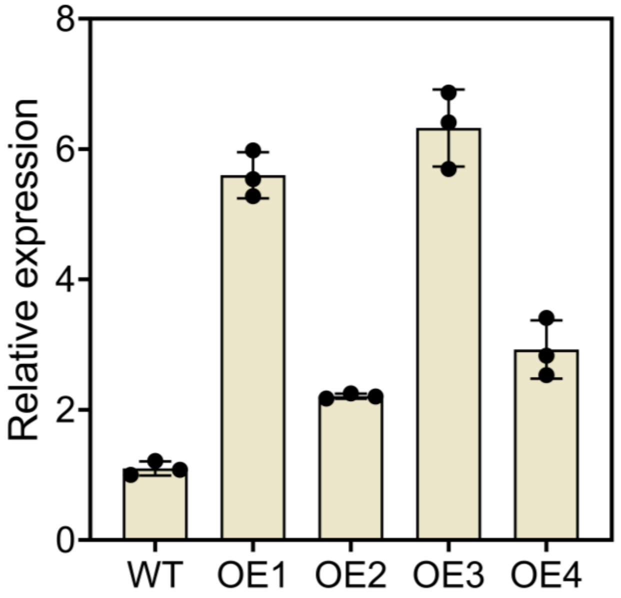

Supplementary Figure S4 Analysis of the Expression of *TaERF3* in WT and *TaERF3*-overexpression wheat. Data are presented as mean ± standard deviation (SD) from three biological replicates (n = 3). Statistical significance was determined using a one-way ANOVA followed by Tukey's HSD post-hoc test for multiple comparisons. Different letters above the bars indicate statistically significant differences ($p < 0.05$).
